# Bone mesenchymal stem cell transplantation via four routes for the treatment of acute liver failure in rats

**DOI:** 10.3892/ijmm.2014.1890

**Published:** 2014-08-11

**Authors:** LIHUA SUN, XIAOTANG FAN, LIJUAN ZHANG, GUIXIU SHI, MAIMAITI AILI, XIAOBO LU, TAO JIANG, YUEXIN ZHANG

**Affiliations:** Department of Hepatology, The First Affiliated Hospital of Xinjiang Medical University, Urumqi, Xinjiang 830054, P.R. China

**Keywords:** acute liver failure, stem cell transplantation, bone mesenchymal stem cells, transplantation method

## Abstract

In the present study, we assessed the efficiency of four BMSC transplantation methods as a therapy for liver failure. A rat model (80 Sprague-Dawley rats) of D-galactosamine (D-gal)/lipopolysaccharide (LPS)-induced acute liver failure (ALF) was established and the rats were divided into 5 groups: a hepatic artery injection group, a portal vein injection group, a vena caudalis injection group, an intraperitoneal injection group and a control group (16 per group). Following transplantation, the liver tissue and blood samples were collected on days 1, 3 and 7, we detected the EdU (5-ethynyl-2′-deoxyuridine)-labeled cells homing to the liver tissue and assessed the proliferating cell nuclear antigen (PCNA) and cysteine-containing aspartate-specific protease (caspase)-3 expression in the liver tissue and detected the levels of stromal cell-derived factor 1 (SDF-1) and hepatocyte growth factor (HGF) in the liver tissues. Compared with the control group, the levels of alanine aminotransferase (ALT) and aspartate aminotransferase (AST) and damage to the liver tissue in the hepatic artery group, the portal vein group and the vena caudalis group improved *in vivo*. The expression of PCNA and HGF in the liver was higher and caspase-3 expression was lower in the hepatic artery injection group, the portal vein injection group and the vena caudalis injection group than that in the intraperitoneal injection and control groups. The EdU-labeled BMSCs were only observed homing to the liver tissue in these three groups. However, no significant differences were observed between these three groups. Liver function in the rats with ALF was improved following BMSC transplantation via 3 endovascular implantation methods (through the hepatic artery, portal vein and vena caudalis). These 3 methods were effective in transplanting BMSCs for the treatment of ALF. However, the selection of blood vessel in the implantation pathway does not affect the transplantation outcome. Transplantation via intraperitoneal injection showed no therapeutic effect in our animal experiments.

## Introduction

Acute liver failure (ALF) is a dramatic but rare clinical syndrome marked by the sudden loss of hepatic function in a person with no prior history of liver disease (1). The course of ALF is variable and the mortality rate is high. Orthotopic liver transplantation (OLT) is the most effective treatment for severe liver injury; however, the widespread clinical application of liver transplantation is restricted by the shortage of available donor livers and multiple post-operative complications (2,3). Due to its potential to promote liver regeneration with fewer complications than the surgical approach, the use of stem cells seems to be a promising method for the treatment of liver diseases (4–6). Sources of exogenous stem/progenitor cells that are currently under investigation in the context of repair of liver injury include embryonic stem cells, bone marrow-derived mesenchymal stem cells (MSCs), fat-derived mesenchymal stem cells, fetal annex stem cells and endothelial progenitor cells (EPCs) (7–10).

MSCs represent an attractive option for successful stem-cell-based therapy for liver diseases due to their ready accessibility, minimal invasiveness and rapid proliferation (11). Furthermore, isolated MSCs are multipotent and can differentiate into multiple lineage cell types, including mesodermal cell lineages, such as osteoblasts, adipocytes, chondroblasts, myocytes and cardiomyocytes, as well as non-mesodermal cells, such as hepatocytes and neurocytes (12).

For therapeutic applications, it is important to understand the potency and possible repair mechanisms of MSCs. Previous studies have demonstrated that MSCs can differentiate into functional hepatic cells and can also produce a series of growth factors and cytokines able to suppress inflammatory responses, reduce hepatocyte apoptosis and enhance hepatocyte functionality (13,14). To date, few comparative studies of different methods for the transplantation of bone marrow MSCs are available. Furthermore, the association between the transplant route and the transplant effect remains unclear. In animal experiments, MSC transplantation typically proceeds via portal vein injection, hepatic artery injection, peripheral vein injection, intraperitoneal injection or local injections into the liver or spleen. In clinical applications, the principal transplantation methods are portal vein injection, hepatic artery injection and peripheral vein injection; however, the optimal method for transplanting MSCs has not yet been identified. Kuo *et al* (11) demonstrated that both MSC-derived hepatocytes and MSCs transplanted through either the intrasplenic or the intravenous route can be engrafted into the recipient liver and differentiate into functional hepatocytes. Intravenous transplantation was found to be more effective in rescuing liver failure than intrasplenic transplantation. Wang *et al* (15) investigated the therapeutic effects of bone mesenchymal stem cells (BMSCs) on liver cirrhosis in rats induced by CCl4 via partial liver BMSC transplant, tail vein transplant, partial liver transplant, spleen transplant and portal vein transplant. Their results indicated that partial liver BMSC transplantation is more effective in liver cirrhosis than other transplantation routes. MSC transplantation via the spleen contributed little to the restoration of liver function as the transplanted cells usually grow in nodules, while MSC transplantation by hepatic multi-site injection involves the risk of damaging important liver vessels and causing severe complications, including hemorrhea and portal hypertension. Xiong *et al* (16) demonstrated that the transplantation of MSCs into rats with cirrhosis via the portal vein, hepatic artery and vena caudalis had similar curative effects. Zhao *et al* (17) indicated that the intravenous injection of MSCs was effective in treating liver fibrosis compared with intrahepatic injection and intraperitoneal injection. Cao *et al* (18) showed that the use of human placenta MSCs (hPMSCs) prolonged the survival time of pigs with ALF and that the left branch of the portal vein inside the liver offered a superior route compared with the jugular vein pathway. Kim *et al* (19) observed that the transplantation of adipose tissue-derived stem cells (ADSCs) into mice with ALF via a peripheral vein (tail vein) resulted in more prominent liver function than via the portal vein and direct liver parenchymal injection. Other studies have investigated transplantation methods in other diseases. For example, Li *et al* (20) investigated the transplantation of human umbilical cord MSCs for the treatment of acute tubular necrosis and showed that the cells can survive in the kidneys, while the benefits of intravenous injection and arterial injection in repairing the kidney were similar. Zonta *et al* (21) investigated the most effective route of administration (intra-arterial vs. intravenous) to achieve immunomodulating effects in experimental rat kidney transplantation and demonstrated that the intra-arterial infusion of MSCs was more effective in controlling acute rejection.

In this study, we compared the therapeutic effects among 4 different protocols for MSC transplantation (hepatic artery injection, portal vein injection, vena caudalis injection and intraperitoneal injection) in the treatment of D-galactosamine (D-gal)/lipopolysaccharide (LPS)-induced ALF. In addition, we aimed to elucidate the possible mechanisms responsible for the different outcomes according to the cell transplant site.

## Materials and methods

### Animals

Male Sprague-Dawley (SD) rats aged 3–4 weeks (weighing 80–120 g) were used as BMSC donors. Male SD rats aged 8 weeks (weighing 250–280 g) were used as BMSC recipients. All the rats were purchased from the Animal Center for Disease Control in Urumqi, China and kept in the animal facility of the First Affiliated Hospital of Xinjiang Medical University, Urumqi, China. All procedures were approved by the Ethics Committee of the First Affiliated Hospital of Xinjiang Medical University (permit no. A-20100723015) in compliance with institutional animal care guidelines.

### Induction of ALF

The rats were injected intraperitoneally to induce ALF with D-gal [1.4 g/kg/per injection *bis in die (*b.i.d.; twice daily), 12-h interval between injections, Sigma-Aldrich Inc., St. Louis, MO, USA] combined with LPS (0.005%, 20 μg/kg, Sigma-Aldrich Inc.). The extent of hepatic damage was evaluated by a biochemical analysis of blood samples and a histological examination of liver tissue taken from the sacrificed rats. The rats were sacrificed using a mixture of amiodarone 2 ml + diazepam 2 ml + atropine 1 ml + physiological saline 5 ml (7.5 ml/kg). Liver tissues were immediately stored at −80°C for molecular detection or fixed in 10% (v/v) formalin for histological and immunohistochemical analyses.

### Isolation and culture of rat BMSCs

BMSCs obtained from SD rats were isolated and cultured according to an established protocol (22). The rats were anesthetized with 8% ketamine (4.0 ml/kg), the femurs and tibiae of the rats were excised and the soft connective tissue was removed. The 2 ends of the femurs and tibiae were excised and the cells of the bone marrow were harvested by flushing the bone marrow cavity with complete culture medium. The extract was filtered using a 200-mesh filter, centrifuged for 5 min at 1,000 × g, resuspended in medium and inoculated into a 25-cm^2^ culture dish containing Dulbecco’s modified Eagle’s medium with low glucose (DMEM-LG; Gibco Corp, Carlsbad, CA, USA) supplemented with 10% fetal bovine serum (FBS; HyClone, Logan, UT, USA), 1% penicillin/streptomycin and 2 mmol/l L-glutamine at 37°C in 5% CO_2_. The medium in the culture dish was replaced with the same volume of fresh culture medium after 48 h, then replaced every 3–5 days when the adherent cells reached 70–80% confluence. Cell collection was completed with 0.25% trypsin/ethylenediaminetetraacetic acid (EDTA; Solarbio Inc, Beijing, China) treatment and subcultured using a 25-cm^2^ culture dish.

### Labeling and tracing of rat BMSCs

The BMSCs (third passage) were collected using 0.25% EDTA and cultured with DMEM medium containing 10% FBS (cell density 5×10^3^/cm^2^). After 24 h, 5-ethynyl-2′-deoxyuridine (EdU; Cell-Light™ EdU Apollo™ DNA *in vivo* Kit, Bo Rui Inc., Guangzhou, China) was added to the medium at a concentration of 10 μM. After 72 h, the cells were washed twice with phosphate-buffered saline (PBS). Approximately 2×10^7^ EdU-labeled BMSCs were subsequently utilized for injection. After transplantation, the tracing of the EdU-labeled BMSCs in the liver tissues was carried out using immunofluorescence staining according to the manufacturer’s instructions. The liver tissues were fixed with methanol, dewaxed and then incubated in 0.5% Triton^®^ X-100 in PBS at room temperature. The tissues were then incubated with an Apollo reaction cocktail for 30 min at room temperature without light, counterstained with Hoechst 33324 reaction cocktail for 30 min at room temperature without light, and imaged under a fluorescence microscope (OlympusBX5l; Olumpus, Tokyo, Japan), as previously described (23).

### General experimental protocols

A total of 80 SD rats with ALF were randomly divided into 5 groups (n=16 in each group) and injected via the hepatic artery (group 1), the portal vein (group 2), the vena caudalis (group 3) and by intraperitoneal injection (group 4). Group 5, in which the rats received the same volume of isotonic saline but no BMSCs via the vena caudalis, served as the controls. At 24 h after the administration of D-gal/LPS, the 0.8 ml EdU-labeled BMSC suspensions (passages 3 to 6, 1.4×10^7^/kg) were injected through the different routes within 15 min. The procedure was performed under general anesthesia. There were 5, 5 and 6 animals per group sacrifice on days 1, 3 and 7 post-implantation, respectively.

### Flow cytometry

The phenotypic profile of the BMSCs was evaluated by flow cytometric analysis (fluorescence-activated cell sorting, Epic XL, Software Expo 32; Beckman Coulter, Fullerton, CA, USA), using CD29, CD90, CD45 and CD11b/c antibodies (BioLegend, San Diego, CA, USA), coupled to either phycoerythrin or fluorescein isothiocyanate.

### Alanine aminotransferase (ALT) and aspartate aminotransferase (AST) levels

Blood samples were obtained from each rat in the 5 groups on days 1, 3 and 7 post-transplantion. The samples were immediately centrifuged at 2,000 × g for 6 min before serum was collected. The ALT and AST levels were measured using a serum multiple automatic biochemical analyzer (HITACHI-7150; Olympus).

### Histological examination

Specimens from the rat livers in all groups were fixed in 4% formaldehyde, embedded with paraffin, sectioned and stained with hematoxylin and eosin (H&E). Pathological findings were assessed according to the Scheuer score by one of the authors blinded to the group allocations.

### Immunohistochemistry for proliferating cell nuclear antigen (PCNA) and cysteine-containing aspartate-specific protease (caspase)-3

Regeneration and apoptotic hepatocytes were identified by immunohistochemistry. According to the manufacturer’s instructions, immunohistochemical staining was performed to evaluate PCNA [fluorescein isothiocyanate-labeled monoclonal mouse clone PC10; LifeSpan BioSciences (LSBio), Seattle, WA, USA] and caspase-3 (fluorescein isothiocyanate-labeled monoclonal rabbit clone CPP32-Ab-4, Thermo Fisher Scientific, Waltham, MA, USA). Positive cells were counted in 10 random visual fields at ×200 magnification for each section, and the number was expressed as the integrated optical density (IOD) value. The sections were examined microscopically for specific staining, and photographs were acquired using a digital image-capture system (Olympus CX40; Olympus, Tokyo, Japan).

### Enzyme-linked immunosorbent assay

Blood was collected from the rats in the 5 different groups at 1, 3 and 7 days post-transplantation. Serum samples were assayed for stromal cell-derived factor 1 (SDF-1) production with an SDF-1 enzyme-linked immunosorbent assay (ELISA) quantification kit (GBD, Santiago, CA, USA) according to the manufacturer’s recommendations.

### Quantitative reverse transcription-polymerase chain reaction (RT-qPCR)

Total RNA was extracted from the liver tissue (50 mg) using TRIzol reagent (Invitrogen, Carlsbad, CA, USA). RNA (5 μg) was reverse-transcribed using the AMV First Strand cDNA Synthesis Kit (Invitrogen) following the manufacturer’s instructions, yielding the complementary DNA (cDNA) template. The cDNA was then amplified by PCR using the primer sequences (ShengGong, Shanghai, China) shown in Table I. After PCR, 10 μl of the reaction mixture were subjected to electrophoresis on a 1% agarose gel. The size of the PCR fragments was estimated using a 100-base-pair ladder. The quantitative PCR amplification and data analysis were performed using fluorescence quantitative PCR (StepOnePlus; Applied Biosystems, Foster City, CA, USA). Primer sequences are listed in Table I.

### Statistical analysis

Data are presented as the means ± standard deviation (SD). Differences in parameters were analyzed using a one-way analysis of variance (ANOVA). All analyses were performed using SPSS version 17.0 statistical software (SPSS Inc, Chicago, IL, USA). Values of P<0.05 were considered to indicate statistically significant differences.

## Results

### Morphology of cultured rat BMSCs

**Figure 1:**
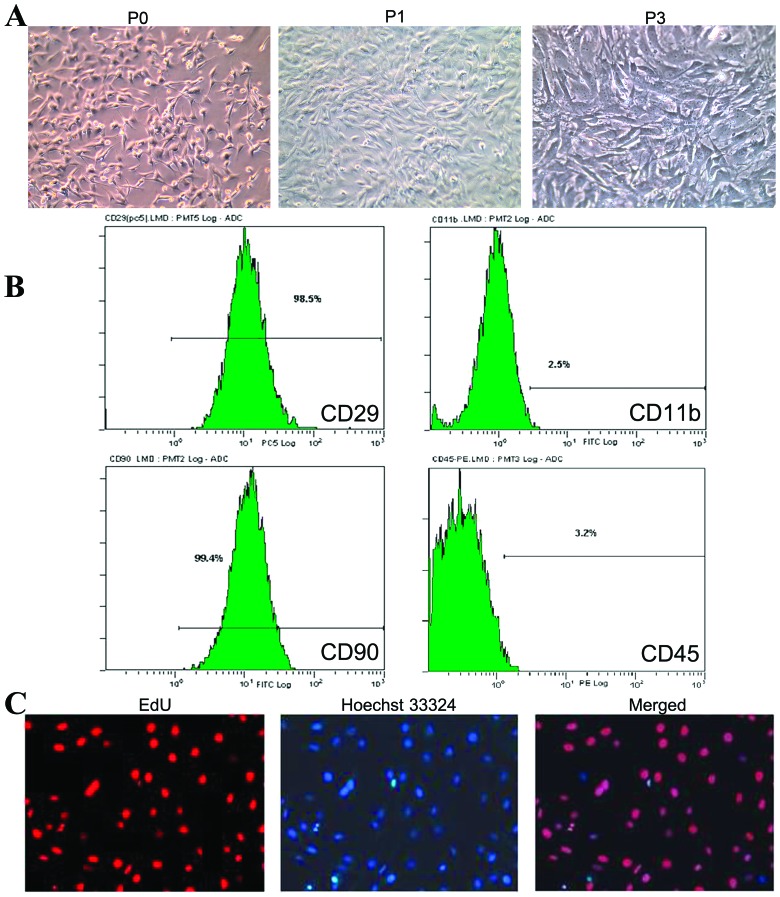
Rat bone mesenchymal stem cells (BMSCs). (A) Morphology of cultured rat BMSCs transplanted using the 3 methods, as shown under a phase-contrast microscope. P0, morphology of primary passage BMSCs (x100 magnification); P1, morphology of BMSCs in first passage (x100 magnification); P3, morphology of BMSCs in third passage (x100 magnification). (B) Immunophenotype of culture-expanded cells. Fluorescence-activated cell sorting (FACS) analysis for MSC markers. The BMSCs were positive for CD29 and CD11b and negative for CD90 and CD45. Scale bars, 100 mm. FITC, fluorescein isothiocyanate; PE, phycoerythrin. (C) EdU-labeled BMSCs under a fluorescence microscope. The nucleus of EdU-positive cells showed intense red fluorescence; Hoechst 33324 staining was observed in the nucleus (blue fluorescence). Nucleus showing EdU and Hoechst 33324 staining (x200 magnification).

The adherent cells adopted a colony-like distribution within 3 days after inoculation into the flask, as shown under a phase-contrast microscope. Typically, approximately 70–90% confluence was reached by days 7–9. Cells of the third passage had a typical spindle-shaped, fibroblastic morphology and good refractivity. The profile of the cells could clearly be seen, but the nuclei were not distinctly visible (Fig. 1A).

### Surface markers of isolated rat BMSCs and EdU-labeling efficiency

The immunophenotype of the culture-expanded cells (passages 3 to 6) was analyzed by flow cytometry. The BMSCs isolated from rat bone marrow were positive for CD29 (98.5%) and CD90 (99.4%) and negative for CD11b (2.5%) and CD45 (3.2%) (Fig. 1B). When the concentration of EdU was 10 μmol/l, the labeling efficiency of the rat BMSCs was 93%. A fluorescence microscope revealed that the nucleus of the EdU-positive cells was intensely red, with Hoechst 33324 staining observed in the nucleus (blue fluorescence). EdU is a nucleus-specific tag, located in the nucleus together with Hoechst 33324 (Fig. 1C).

### Effect of BMSC transplantation on liver function in rats with ALF

The levels of ALT and AST gradually decreased after transplantation in all the groups. On day 7 post-implantation, the levels of ALT and AST in the hepatic artery injection group, portal vein injection group and vena caudalis injection group were lower than those in the control group (P<0.05). Compared with the control group, there was no statistically significant difference in ALT and AST levels in the intraperitoneal injection group on day 7 post-operation (P>0.05) (Table II).

**Figure 2:**
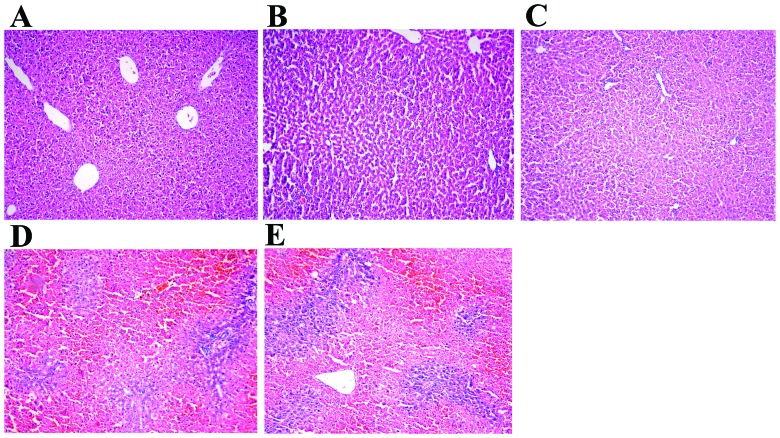
Comparison of liver histological staining of bone mesenchymal stem cells (BMSCs) transplanted with different methods. (A) BMSCs transplanted via hepatic artery injection; (B) BMSCs transplanted via portal vein injection; (C) BMSCs transplanted via vena caudalis injection; (D) BMSCs transplanted via intraperitoneal injection; (E) control injection [mean Scheuer score (A) 2; (B) 2; (C) 2; (D) 4; (E) 4]. All images are at ×100 magnification.

Three days after transplantation, histological staining indicated that the sham (isotonic saline)-injected rats with D-gal/LPS-induced ALF suffered serious inflammation. Non-normal liver lobules had dispersed throughout the liver sinus, hepatic sinusoid had dissociated, hepatocytes had diffuse necrosis, and a large number of inflammatory cells had infiltrated into the necrotic areas. In the hepatic artery, portal vein and vena caudalis injection groups, histological staining indicated that hepatocyte edema, degeneration and necrosis were improved, while inflammatory cell infiltration was significantly reduced. The intraperitoneal transplantation group displayed schistic necrosis, eosinophilic changes and infiltration of inflammatory cells (Fig. 2).

### SDF-1 levels in serum and liver tissue

**Figure 3:**
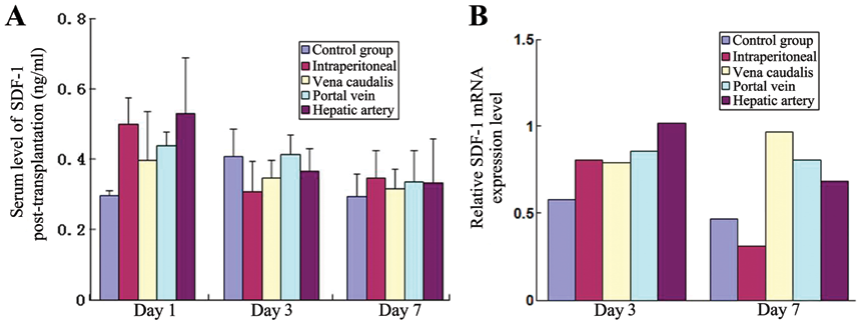
(A) Serum levels of stromal cell-derived factor 1 (SDF-1) post-transplantation using the different transplantation methods. (B) Comparison of SDF-1 mRNA expression following the transplantation of the BMSCs with the different methods.

The levels of SDF-1 in serum as assessed by ELISA and the expression of SDF-1 mRNA in the liver tissue as measured by RT-qPCR revealed no significant differences between the 5 groups (P>0.05) (Fig. 3).

### Homing of BMSCs post-implantation

**Figure 4:**
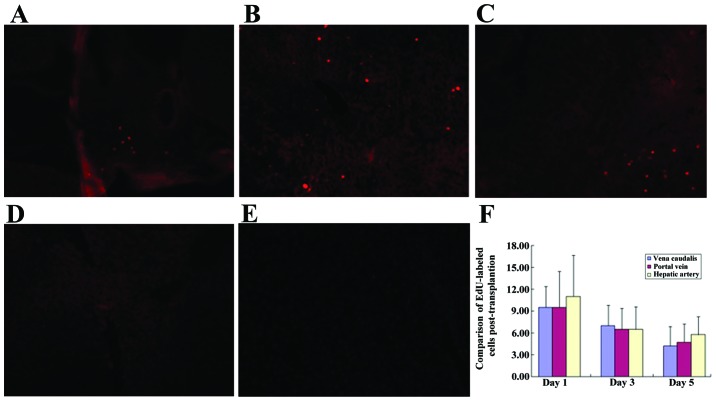
Homing of BMSCs at 3 days post-implantation (x200 magnification). Cell location was observed under a confocal microscope shown by red fluorescence. Transplantation via (A) hepatic artery; (B) portal vein; (C) vena caudalis; (D) intraperitoneal injection; and (E) control group. (A–C) Red fluorescence indicated that the cells were in the liver tissue of the recipient mice, indicating the existence of EdU-positive cells. (F) Comparison of EdU-labeled cells post-transplantation.

The homing of BMSCs in the liver tissues was examined using immunofluorescence on days 1, 3 and 7 following BMSC transplantation. Transplanted BMSCs were not observed in the intraperitoneal injection group or the control group. Labeled transplanted cells were observed in the hepatic artery, the portal vein and the vena caudalis injection groups on day 1. Labeled transplanted cells were predominantly distributed in the hepatic sinusoid. The numbers of EdU-labeled cells were 4.25±2.63/high power lens (HP), 7.00±2.83/HP and 9.5±2.89/HP in the hepatic artery, the portal vein and the vena caudalis injection groups, respectively. On day 3 post-transplantation, the numbers of positive cells in the hepatic artery, the portal vein and the vena caudalis injection groups were 4.75±2.5/HP, 6.5±2.89/HP and 9.50±4.95/HP, respectively. The positive cells were predominantly distributed around the damaged live tissue. On day 7, signs of the transplanted cells were still present in the hepatic artery, portal vein and vena caudalis injection groups (5.75±2.50/HP, 6.5±3.11/HP, 11.00±5.66/HP, respectively). There were no statistically significant differences in the number of homing transplanted cells between these 3 groups (P>0.05) (Fig. 4).

### Expression of PCNA in liver tissue

**Figure 5:**
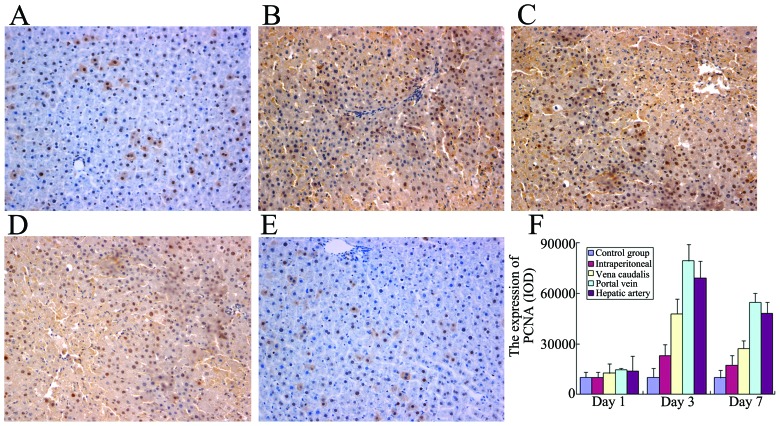
Expression of proliferating cell nuclear antigen (PCNA) in liver tissue (3 days post-transplantation) (x200 magnification) (A) Control group; transplantaion via (B) hepatic artery; (C) portal vein; (D) vena caudalis; (E) intraperitoneal injection. PCNA-positive cell staining in the nucleus was brown-yellow and had a fine granular appearance. (F) Comparison of the expression of PCNA post-transplantation.

The expression of PCNA in the liver tissue was detected on days 1, 3 and 7 by immunohistochemical staining with a monoclonal antibody to PCNA. PCNA-positive cell staining in the nucleus was a brown-yellow color and had a fine granular appearance. On day 3 after transplantation, there was a strong expression of PCNA in the treatment groups. On day 7, PCNA staining levels were weaker than the levels on day 3. At days 3 and 7 after transplantation, PCNA was predominantly expressed in the hepatocytes and bile duct cells. The PCNA staining levels in the hepatic artery, the portal vein and the vena caudalis injection groups were higher than the levels in the intraperitoneal injection group and the control group (P<0.05). There were no significant differences in PCNA staining levels between the intraperitoneal injection group and the control group (P>0.05), or between the hepatic artery, the portal vein and the vena caudalis injection groups (Fig. 5).

### Expression of caspase-3 in liver tissue

**Figure 6:**
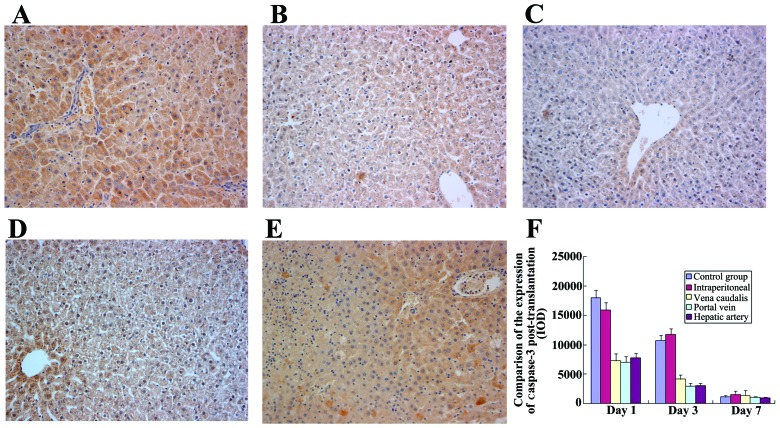
Expression of caspase-3 in liver tissue (3 days post-transplantation) (x200). Caspase-3 positive cells were shown as brown granules, mainly expressed in the portal area around the cytoplasm of the hepatocytes, with low levels of expression in the nucleus. (A) Control group; transplantation via (B) hepatic artery; (C) portal vein; (D) vena caudalis; (E) intraperitoneal injection. (F) Comparison of the expression of caspase-3.

Caspase-3-positive cells were visible as brown granules, predominantly expressed in the portal area around the cytoplasm of the hepatocytes, with some expression in the nucleus. In each group, caspase-3 expression was strong on day 1 post-transplantation, weaker on day 3, and almost entirely absent on day 7. On days 1 and 3, caspase-3 expression in the hepatic artery, the portal vein, and the vena caudalis injection groups was lower than that in the intraperitoneal injection group and the control group (P<0.05). Caspase-3 expression showed no statistically significant differences between the intraperitoneal injection group and the control group (P>0.05), or between the hepatic artery, portal vein and vena caudalis injection groups (P>0.05) (Fig. 6).

### Hepatocyte growth factor (HGF) mRNA level in liver tissue

**Figure 7:**
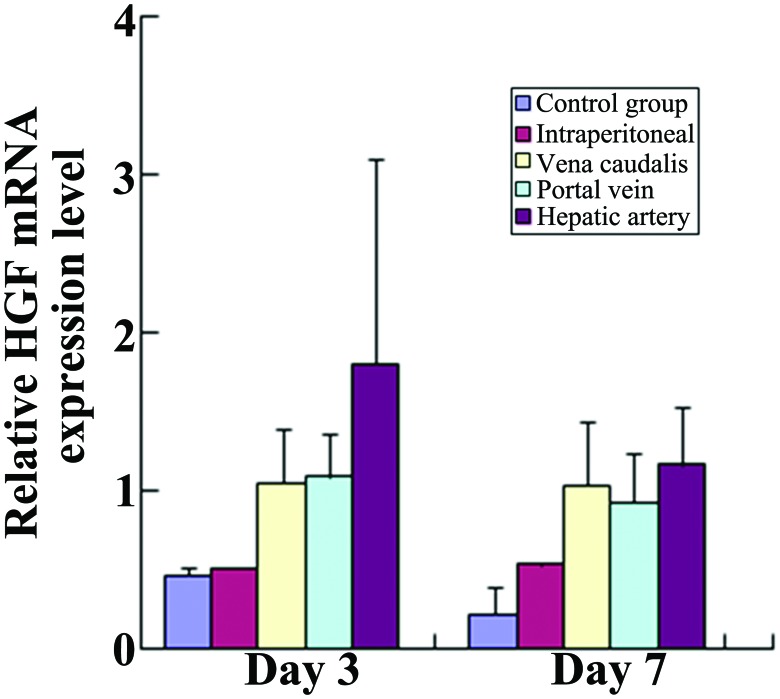
Comparison of hepatocyte growth factor (HGF) mRNA expression following transplantation using the different methods.

On day 3 post-transplantation, the expression of HGF in the 4 treatment groups was significantly higher than that in the control group (P<0.05). However, the expression of HGF in the intraperitoneal injection group was lower than that in the vena caudalis injection group, the portal vein injection group, and the hepatic artery injection group (P<0.05). There were no statistically significant differences between the vena caudalis injection group, the hepatic artery group, and the portal vein groups (P>0.05). On day 7 post-transplantation, the expression of HGF in the vena caudalis injection group, the portal vein group and the hepatic artery group was higher than that in the intraperitoneal injection group and the control group. The difference between the intraperitoneal injection group and the control group was not statistically significant (P>0.05). There were no significant differences between the vena caudalis, the hepatic artery and the portal vein injection groups (P>0.05) (Fig. 7).

## Discussion

ALF is the clinical manifestation of sudden and severe hepatic injury and arises from many causes, such as viruses, drugs and poisons. Due to the abrupt loss of hepatic metabolic and immunological function, the condition leads to hepatic encephalopathy, coagulopathy and in many cases, progressive multi-organ failure (24). Although uncommon, this critical illness occurs mostly in young adults and is associated with high mortality and resource costs. In many countries it is the most frequent indication for emergency liver transplantation (25). The current clinical treatment for liver failure includes medical drugs, artificial liver support treatment, liver transplantation and stem cell transplantation (5,26–28). Among these methods, OLT represents the most suitable therapeutic option for patients with hepatic failure; however, the speed of disease progression, as well as the variable course of ALF and organ shortages limits its use (29). Cell-based therapy has been proposed as a potential alternative to OLT. Allogeneic hepatocyte transplantation has been reported for the treatment of ALF (30,31). However, immune rejection and hepatocyte disorder in *in vitro* cultures have provided obstacles to the widespread use of this type of therapy (32,33).

Given this background, a growing enthusiasm has greeted the development of stem-cell-based therapies for liver diseases. The application of bone marrow MSC transplantation for ALF has been a research hotspot over recent years (19).

MSCs are adherent, fibroblast-like, pluripotent and non-hematopoietic progenitor cells. Numerous studies have demonstrated that MSCs have a high degree of plasticity, as they differentiate into cells of the mesenchymal lineage, but they can also differentiate into neurons, splenocytes and various epithelial cells, including liver, lung, intestinal and kidney cells (34,35). Recent experimental studies have shown the successful application of MSC transplantation in the treatment of fulminant hepatic failure (FHF), end-stage liver disease (ESLD) and inherited metabolic disorders (IMDs) (36–38). According to the International Society for Cellular Therapy (ISCT), MSCs are defined by their expression of CD105, CD73 and CD90 and the lack of expression of CD45, CD34, CD14 or CD11b, CD79a or CD19 and human leukocyte antigen (HLA)-DR (39). In this study, BMSCs were isolated and cultured from SD rats. The immunophenotype of the BMSCs was evaluated by fluorescence-activated cell sorting. More than 98% of the cells stained positive for CD29 and CD90, which is in accordance with the diagnostic characteristics of MSCs. The cells stained negative for CD11b and CD45, which are indicative of neutrophilic granulocyte and hematopoietic cell lines. These results demonstrate that the cultured cells derived from the rat bone marrow consisted of more than 98% MSCs, which is consistent with the results from previous studies (40–42).

Selecting an appropriate MSC transplantation route is vital for cell survival, the induction of cell differentiation and the restoration of liver function. In this study, we injected BMSCs via 4 different routes. Liver function was elevated in the hepatic artery group, the portal vein group and the caudal vein group compared with the control group 7 days post-implantation. There was no significant difference between the intraperitoneal injection group and the control group. Same changes were observed in histological staining. This indicates that hepatocyte edema, degeneration and necrosis were improved, while inflammatory cell infiltration was significantly reduced in the hepatic artery group, the portal vein group and the caudal vein group. Furthermore, these differences had occurred by 3 days post-implantation. These results confirm that BMSCs have a therapeutic effect in the treatment of liver failure.

To further compare the functional restoration of MSCs following hepatic artery, portal vein and caudal vein injection, the levels of ALT and AST were measured, and revealed no differences between these groups. Furthermore, no differences were observed in the extent of histological improvement between these groups. These results indicate that the implantation route may impact on the curative effect of implantation; the endovascular injection of BMSCs provides better treatment than extravascular injection modalities.

SDF-1 is a micro-molecular protein, exhibiting a variety of biologic activities. It has been shown that SDF-1 can promote BMSC homing to the injured livers of mice (43). SDF-1 can also act as a chemoattractant to promote the migration of stem cells (44) and to strengthen their locomotory capacity (45). When stem cells migrate to the target tissue, SDF-1 facilitates their adhesion to fibrinogen, fibronectin, interstitium and endotheliocytes. In this study, the level of SDF-1 in serum and the expression of SDF-1 mRNA in the liver tissue were similar, indicating that the extent of chemotaxis was the same in all the rats with ALF. However, labeled transplanted cells were only observed in the hepatic artery injection group, the portal vein injection group and the vena caudalis injection group, and not in the intraperitoneal injection group or the control group. These results confirm the effect of an endovascular injection of BMSCs in promoting BMSC homing to the injured livers. However, our study did not show superiority among the hepatic artery injection group, the portal vein injection group, or the vena caudalis injection group. In addition, immunohistochemistry of the liver sections for PCNA expression revealed that the PCNA staining levels in the hepatic artery, the portal vein and the vena caudalis injection group were higher than the levels in the intraperitoneal injection group or the control group. The level of HGF mRNA in the liver, according to RT-qPCR, also showed the same pattern (46). These results indicate that the endovascular injection of BMSCs may promote hepatocyte regeneration, as also previously demonstrated (47).

In the early stages of post-implantation, caspase-3 expression in the hepatic artery, the portal vein and the vena caudalis injection group was lower than that in the intraperitoneal injection group and the control group. However, intraperitoneally-injected BMSCs were not a sufficiently effective treatment when compared with the BMSCs in the endovascular injection groups. These results indicate that an endovascular injection of BMSCs has profound inhibitory effects on hepatocellular death, and leads to reduced hepatocyte apoptosis, enhanced liver regeneration and an increased number of proliferating hepatocytes. However, this study did not show superiority among the hepatic artery injection group, the portal vein injection group, or the vena caudalis injection group.

Three factors affected the homing of stem cells in the liver: i) the type and severity of liver damage; ii) the expression of chemokines prompting the homing of stem cells to the damaged liver; and iii) the number of bone marrow stem cells in the circulation. It is well known that chemokines are released after tissue damage and that the migratory direction of stem cells follows the chemokine concentration gradient. The increase in the inflammatory chemokine concentration at the site of inflammation is a key mediator of MSC trafficking to the site of injury (46,48,49). MSCs have an inherent chemotaxis ability to home in to sites of inflammation (50). MSCs express the SDF-1 chemokine receptor [chemokine (C-X-C motif) receptor 4, CXCR4], while the SDF-1/CXCR4 biological axis stimulates the recruitment of progenitor cells to the site of tissue injury (51–54). BMSCs can migrate across endothelial cell layers, attracted to injury tissue and be retained in the ischemic tissue, but not in the remote or intact tissue. Transplantation via an endovascular approach can ensure that stem cells in the blood respond to the concentration gradient of chemotaxis and migrate to the damaged liver tissue. Although the hepatic artery, portal vein and caudal vein have different degrees of influence on liver hemodynamics, and while each involve different homing distances, the different endovascular approaches did not affect the chemotaxis or the homing of stem cells in our study. Due to a deficiency in stem cell homing, the BMSC transplantation via intraperitoneal injection had no therapeutic effect on ALF in rats.

In conlusion, following BMSC transplantation, liver function in the rats with ALF was improved by hepatic artery injection, portal vein injection and vena caudalis injection. The extent of damage in the liver pathology was also reduced. At the same level of the chemotactic factor, SDF-1, the 3 endovascular graft methods (hepatic artery, portal vein and vena caudalis) showed a benefit in terms of the BMSCs homing to the damaged liver tissue, enhancing hepatocyte proliferation and inhibiting liver cell apoptosis; all 3 methods were an effective route for the transplantation of BMSCs for the treatment of ALF. However, the selection of blood vessel as a migration path does not affect the transplantation result. The intraperitoneal injection as a transplantation route showed no therapeutic effect in our animal experiments.

## Figures and Tables

**Table I:** Gene primers used for detection.

Gene	Sense	Antisense
β-actin	ACGTTGACATCCGTAAAGACC	GCCACCAATCCACACAGAGT
SDF1	CATCAGTGACGGTAAGCCAG	CACAGTTTGGAGTGTTGAGGAT
HGF	ATGACATCACTCCCGAGAACTT	GAGAGCAGTAACCAACTCGGAT

**Table II:** Comparison of liver function post-transplantation (IU/l).

	Day 1		Day 3		Day 7	
			
Injection group	ALT	AST	ALT	AST	ALT	AST
Control group	55.4±9.9	126.4±20.2	45.9±10.4	128.8±18.0	57.0±13.4	137.3±36.9
Intraperitoneal	35.7±0.9^a^	109.7±7.9^a^	42.0±10.3^a^	97.0±25.1^a^	44.3±7.3^a^	96.4±24.9^a^
Vena caudalis	52.0±10.7^a^	135.6±16.0^a^	46.1±8.4^a^	97.3±22.5^a^	31.8±4.1^b^	72.0±6.4^b^
Portal vein	60.0±15.6^a^	277.0±108.9^a^	31.8±25.8^a^	118.5±97.3^a^	30.0±6.8^b^	74.3±7.7^b^
Hepatic artery	94.5±61.5^a^	231.0±17.0^a^	19.3±1.3^a^	54.3±6.0^a^	21.5±2.4^b^	63.3±6.1^b^

ALT, alanine aminotransferase; AST, aspartate aminotransferase;

a Compared with the control group P>0.05;

b compared with the control group P<0.05.
